# Darier disease is associated with neurodegenerative disorders and epilepsy

**DOI:** 10.1038/s41598-024-57779-4

**Published:** 2024-03-26

**Authors:** Philip Curman, William Jebril, Henrik Larsson, Etty Bachar-Wikstrom, Martin Cederlöf, Jakob D. Wikstrom

**Affiliations:** 1 Dermatology and Venereology Division, Department of Medicine (Solna), Karolinska Institutet, Stockholm, Sweden; 2 Dermato-Venereology Clinic, Karolinska University Hospital, Eugeniavägen 3, 17164 Stockholm, Sweden; 3 Department of Medical Epidemiology and Biostatistics, Karolinska Institutet, Stockholm, Sweden; 4 School of Medical Sciences, Faculty of Medicine and Health, Örebro University, Örebro, Sweden

**Keywords:** Epidemiology, Skin diseases, Medical genetics

## Abstract

Darier disease (DD) is a rare monogenetic skin disorder with limited data on its potential association with neurological disorders. This study aimed to investigate the association between DD and neurological disorders, specifically Parkinson's disease, dementias, and epilepsy. Using Swedish national registers in a period spanning between 1977 and 2013, 935 individuals with DD were compared with up to 100 comparison individuals each, randomly selected from the general population based on birth year, sex, and county of residence at the time of the first diagnosis of DD. Individuals with DD had increased risks of being diagnosed with Parkinson's disease (RR 2.1, CI 1.1; 4.4), vascular dementia (RR 2.1, CI 1.0; 4.2), and epilepsy, (RR 2.5, CI 1.8; 3.5). No association of DD with other dementias were detected. This study demonstrates a new association between DD and neurodegenerative disorders and epilepsy, underlining the need for increased awareness, interdisciplinary collaboration, and further research to understand the underlying mechanisms. Early identification and management of neurological complications in DD patients could improve treatment strategies and patient outcomes. The findings also highlight the role of SERCA2 in the pathophysiology of neurological disorders, offering new targets for future research and potentials for novel treatments.

## Introduction

Darier disease (DD) is an autosomal dominant genodermatosis characterized by keratotic papules and plaques, malodorous skin, nail abnormalities, and mucosal changes^1^. DD is caused by mutations in the *ATP2A2* gene, which encodes the sarco/endoplasmic reticulum Ca^2+^-ATPase isoform 2 (SERCA2) pump^2^. SERCA2 is ubiquitously expressed and important for maintaining Ca^2+^ homeostasis in the endoplasmic reticulum^3,4^. Dysfunction of SERCA2 leads to abnormal intracellular Ca^2+^ levels and impaired cell adhesion, ultimately resulting in the characteristic skin manifestations of DD. Being a rare disorder, the estimated prevalence of DD is 1 in 30,000–100,000 individuals^5^.

Although DD is traditionally considered to be a skin disorder, several studies have suggested potential links between DD and psychiatric disorders^6,7^. In a descriptive study of 163 DD patients, a larger occurrence of epilepsy than the population baseline has been seen^1^. The evidence for an association with neurological disorders is, however, lacking. The *ATP2A2* gene is expressed in various regions of the brain, and the role of SERCA2 dysfunction in the neurobiology and pathophysiology of several neurological disorders is well documented^3^. Given these findings, this study aimed to investigate the plausible association between DD and common neurological disorders such as Parkinson's disease (PD), vascular dementia, Alzheimer’s disease (AD), other dementias, and epilepsy.

## Results

Individuals with DD had an increased risk of being diagnosed with PD (RR 2.1, CI 1.1; 4.4) and vascular dementia (RR 2.1, CI. 1.0; 4.2), whereas no association with other dementias could be confirmed (RR 1.1, CI 0.7; 2.0). Due to insufficient statistical power, the association of DD with AD could not be assessed. Finally, there was an increased risk of epilepsy in individuals with DD (RR 2.5, CI 1.8; 3.5). Risk ratios adjusted for common and impactful vascular risk factors did not more than marginally differ from unadjusted risk ratios regarding PD and epilepsy, whereas the adjusted risk ratio for vascular dementia turned insignificant (RR 1.5, CI 0.8; 4.9). All results are presented in Table 1.

**Table 1:** Risk of neurodegenerative disorders and epilepsy in individuals with Darier disease.

	Parkinson’s disease			Vascular dementias			Alzheimer’s disease		Other dementias			Epilepsy		
	N (%)	RR (CI)	RR^a^ (CI)	N (%)	RR (CI)	RR^a^ (CI)	N (%)	RR (CI)	N (%)	RR (CI)	RR^a^ (CI)	N (%)	RR (CI)	RR^a^ (CI)
Individuals with Darier disease, n = 935	8 (0.9)	2.1 (1.1;4.4)	2.0 (1.0;5.1)	8 (0.9)	2.1 (1.0;4.2)	1.5 (0.8;4.9)	2 (0.2)	n/a	14 (1.5)	1.1 (0.7;2.0)	1.0 (0.6;2.2)	37 (4.0)	2.5 (1.8;3.5)	2.4 (1.6;4.1)
Comparison individuals without Darier disease, n = 93,487	379 (0.4)			403 (0.4)			732 (0.8)		1239 (1.3)			1522 (1.6)		

Individuals with Darier disease are compared with matched comparison individuals without Darier disease. Associations are risk ratios (RR) with corresponding 95% confidence intervals (CI). RR^a^: risk ratios adjusted for common vascular risk factors: stroke, myocardial infarction, heart failure, essential and secondary hypertension, type 1 and 2 diabetes, and hyperlipidemias.

## Discussion

Our findings revealed for the first time that individuals with DD had an increased risk of PD, vascular dementia, and epilepsy. This suggests a potential connection between DD and these neurological disorders and constitutes direct human evidence for the role of SERCA2 in neurological disease. Although the underlying mechanisms remain unclear, several hypotheses can be proposed. For instance, the *ATP2A2* gene, which is causal in DD, is also involved in Ca^2+^ signaling in neurons^8^. Dysregulation of Ca^2+^ signaling has been implicated in the pathophysiology of various neurological disorders, including PD and epilepsy^9^. No association between DD and AD was seen due to low insufficient statistical power. Considering that AD is the most prevalent neurodegenerative disorder, a reasonable conclusion is that there might indeed be no association. Further, the association with vascular dementia lost significance when adjusting for a large array of vascular risk factors, tempering this association.

SERCA2 is a ubiquitously expressed protein, meaning that the *ATP2A2* mutations give rise to lower functioning levels of SERCA2 in all tissues, not just the skin^4^. Given the important role of SERCA2 in the brain, it is reasonable that individuals with DD also display increased risks of neurological disorders, as shown in this investigation. Several recent studies have given evidence of DD being a disorder with significant comorbidities including heart failure, diabetes, and cognitive dysfunction^10–12^. This further implies the ubiquitous nature of SERCA2 dysfunction in DD and broadens the understanding of DD as a multisystem condition.

Moreover, the genetic background of DD may predispose individuals to a higher risk of developing neurological disorders due to shared genetic factors or pleiotropic effects^13^. It is currently unknown whether pleiotropic effects or interactions of *ATP2A2* mutations with other specific constellations of genes can explain the associations seen. Furthermore, the common embryological origin of both skin and nervous tissue from the ectoderm might imply a shared pathophysiological role. Future research should focus on identifying potential genetic modifiers or interactions that could contribute to the observed associations. An alternative speculative explanation could be that individuals with DD may experience increased exposure to risk factors for developing neurological disorders throughout their lives.

### Strengths and limitations

The main strengths of this study are the rigorous methodology with a longitudinal design and incidence density sampling scheme with a 1:100 matching ratio, allowing for a robust estimation of the association between DD and neurological disorders. The sample size of 935 individuals with DD is uniquely large given the rarity of DD, further increasing statistical power and the reliability of the findings. Lastly, Swedish nationwide registers are comprehensive and of very high quality.

The lack of association between DD and other dementias may be due to the limited sample size and the need for further research to explore this relationship. It is also important to note that the study was unable to provide an estimation of the association with Alzheimer's disease due to insufficient power since only two individuals with DD in our cohort had such a diagnosis. One limitation of this study is the reliance on register-based data, which may be subject to diagnostic misclassification. However, a validation study has shown that diagnoses of chronic somatic diseases in the NPR, such as DD, are generally valid^14^, and assigned by specialist physicians in their respective fields.

## Conclusions

This study provides evidence for an association between DD and certain neurological disorders, including PD, vascular dementia, and epilepsy. Clinicians treating patients with DD should be aware of the potentially increased risk of neurological disorders in this population. Early identification of neurological complications in individuals with DD may enable improved management and treatment strategies, ultimately enhancing patient outcomes. The observed associations underscore the importance of interdisciplinary collaboration in the care of patients with DD and co-occurring neurological disorders. These findings also highlight the need for further research to better understand the underlying mechanisms linking DD and neurological disorders. Lastly, further light is shed on the role of SERCA2 in brain pathophysiology, providing new targets for future research and highlighting the potential for therapeutic interventions.

## Methods

### Ethics

This study was approved by the Swedish Ethical Review Authority. All methods were carried out in accordance with relevant guidelines and regulations.

### Study population

We identified a total of 935 individuals with DD and matched them with up to 100 comparison individuals each, all derived and randomly selected from the general population. Matching was done for birth year, sex, and county of residence at the time of the first DD diagnosis of the index person, i.e., an incidence density sampling scheme. The study period spanned between 1977 and 2013.

### Data sources

We performed a longitudinal cohort study based on the Swedish Total Population Register, which holds demographic information on all individuals registered as Swedish inhabitants since 1968, including immigrants and The National Patient Register (NPR), which includes discharge diagnoses for inpatient care since 1973 and for outpatient care since 2001^15^. All diagnoses were assigned by the treating medical doctor according to International Classification of Diseases, Ninth and Tenth Revision (ICD-9/10). The linkage between registers was made possible by the unique personal identification number, which is assigned to all individuals born in Sweden since 1932, and to immigrants at their arrival to Sweden.

### Study variables

DD was the exposure in the study and defined as a diagnosis of Q82.8E (ICD-10) or 757D (ICD-9) in the NPR. PD, vascular dementia, AD, other dementias, and epilepsy were the outcomes of the study and defined as (1) G20 (ICD-10) or 332A (ICD-9) for PD, (2) F01 (ICD-10) or 290E (ICD-9) for vascular dementias, (3) F00 and G30 (ICD-10) or 290A-B and 331A (ICD-9) for AD, (4) F02, F03, F051, G311 and G318A (ICD-10) or 290W, 290X, 294B, 331B, 331C and 331X (ICD-9) for other dementias, and (5) G40-41 (ICD-10) or 345 (ICD-9) for epilepsy. Vascular risk factors in the adjusted analysis were defined as I63 (ICD-10) or 433, 434, and 436 (ICD-9) for stroke, I21 (ICD-10) or 410 (ICD-9) for myocardial infarction, I50 (ICD-10) or 428 (ICD-9) for heart failure, I10.9 (ICD-10) or 401 (ICD-9) for essential hypertension, I15.9 (ICD-10) or 405 (ICD-9) for secondary hypertension, E10 (ICD-10) or 250 (ICD-9) for diabetes type 1, E11 (ICD-10) or 250 (ICD-9) for diabetes type 2, and E78.0, 1, 2, 5 (ICD-10) or 272A, B, C, E (ICD-9) for hyperlipidemias.

### Statistical analysis

Conditional logistic regression analysis was performed to assess the associations between exposure and outcomes, with results expressed as odds ratios and corresponding 95% confidence intervals. SAS 9.3 software (SAS Institute, Cary, NC) was used for all analyses. As a result of the incidence density sampling, all odds ratios can be interpreted as risk ratios (RR).

## Data Availability

The datasets used and/or analysed during the current study available from the corresponding author on reasonable request.
